# Exploring Trends in Immediate Postresidency Career Paths: A Multi-year Analysis of Plastic Surgery Resident Graduates Across the United States

**DOI:** 10.1093/asjof/ojad115

**Published:** 2023-12-24

**Authors:** Mason J Horne, Stephanie M C Bray, Benjamin J Schalet, Dzifa S Kpodzo

## Abstract

**Background:**

Plastic surgery is one of the most diverse specialties in medicine. Because of the competitiveness of plastic surgery residency, applicants are entering the field with increased experience and more developed interests in specific specialties. Programs and prospective applicants may find it beneficial to know trends in the career paths of recent graduates.

**Objectives:**

To identify trends in postresidency career paths for plastic surgery graduates.

**Methods:**

Data from all integrated plastic surgery residency programs were analyzed from 2013 to 2022. Eighty-eight residency programs were analyzed for review. Residency websites were the primary source of data. Postresidency career paths were categorized into subspecialty fellowships, academic practice, or private practice. Secondary data included program rank, size of the program, associated fellowship program, associated independent program, and program location.

**Results:**

Seventy-three programs met the inclusion criteria. Private practice was the most common immediate postgraduation path. Microvascular and aesthetic fellowships demonstrated maximum growth in the last 10 years, followed by hand fellowships. Programs ranked in the top 25 by Doximity reputation were significantly associated with graduates going into craniofacial (*P* = .05) and microvascular fellowship (*P* = .021), and immediate academic practice (*P* = .011). Lower-ranked programs were correlated with higher levels of graduates entering directly into private/community hospital practice (*ρ* = 0.327).

**Conclusions:**

Life after residency is a necessary consideration for training physicians. Understanding trends in postresidency career paths could help programs and prospective applicants make more informed decisions on what programs may offer the best opportunities to pursue their desired career path.

**Level of Evidence: 5:**

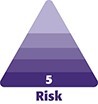

Residency training is a critical period during a physician's career. There are many factors that future physicians must consider when deciding where to pursue their residency training. Certain physician career paths require more in-depth planning when considering residency training. Plastic surgery, like many other surgical specialties, encompasses a broad range of specialized subfields, providing a broad spectrum of opportunities for trainees to pursue. Compared with other specialties, plastic surgery residency requires a longer training period. There are 2 main routes to becoming a plastic surgeon. An integrated residency pathway, which involves a 6-year training period focusing specifically on plastic surgery, and an independent pathway, which requires prior completion of another surgical residency (typically general surgery), followed by a 3-year plastic surgery training period.

Interest in plastic surgery continues to rise, with the specialty consistently ranked as one of the most selective residency fields.^1^ Plastic surgery applicants consistently achieve the highest tiers among United States Medical Licensing Exam scores, class rank, Alpha Omega Alpha honor society membership percentage, and number of research experiences (manuscript publications, research presentations, research abstracts), making these candidates highly desirable for residency programs.^2^ Factors that play a role in residency program attractiveness, such as perception of resident happiness, high operative volume, faculty mentorship, and strong research infrastructure, have been described.^3^ Another crucial consideration that may play a role in how medical students choose a residency program is the immediate graduation paths of the residents at a particular program. Following residency, graduates may choose to work in a private/community hospital setting, an academic setting, a military setting, or a specialized fellowship program.

In recent years, the field has undergone significant growth and expansion due to advancements in surgical techniques, increased patient demand, and emerging subspecialties. These subspecialties include aesthetic, burn, craniofacial, gender-affirming, hand and upper extremity, and microvascular. The continued development of subspecialties within the field has led to the growth of plastic surgery fellowships. Fellowships provide an intense training period in a focused area, allowing for graduates to advance in areas that may be lacking in residency training and to be considered specialists.^4^

Through mentorship and previous clinical and research experiences, residency candidates may already have a career goal in mind when applying for plastic surgery residency training. Currently, there is a paucity of literature describing trends in immediate postresidency pathways of recent graduates of plastic surgery residency. The aim of this study is to analyze trends in immediate postgraduation career paths within plastic surgery residency programs in the United States over the last 10 years.

## METHODS

### Data Collection

This research was reviewed and determined exempt by the institutional review board at Albany Medical College. Data from all integrated residency programs were analyzed from January 2013 to December 2022. The data collection process was completed from May 2023 to June 2023. A total of 88 residency programs were analyzed to compile the dataset. Residency program websites were used as the primary source for data collection. Most residency websites included an alumni page that displayed the immediate postresidency career path of graduates. For the minority of programs that did not include an alumni page, a combination of Doximity (San Francisco, CA) and program Instagram (Meta, Menlo Park, CA) pages were used. If an alumni's postresidency career path was not specified, their clinician biography was analyzed on their current practice, personal website, or their LinkedIn (Microsoft, Sunnyvale, CA) page. If there were discrepancies in those sources, the personal website information was used. If there was no personal website available, and there was a discrepancy between current practice biography and LinkedIn, the current practice biography was used. This strategy has been outlined in multiple recent plastic surgery research studies.^5,6^

### Outcome Variables

Postresidency career paths were divided into 10 categories: aesthetic, burn, craniofacial, gender-affirming, hand/upper extremity, microvascular fellowship, academic, private practice/community hospital (PPCH), military practice, and other. Included in the hand/upper extremity category were orthopedic hand fellowship and hand/peripheral nerve microsurgical fellowship. Included in “other” were trauma, neuroplastic, and postresidency research fellowship. Secondary data collected included the rank of the program, the size of the program, if programs had associated fellowships, if programs had associated independent programs, and the location of the program (Northeast, South, West, Midwest). Program rank was gathered from the Doximity reputation ranking system (accessed June 28, 2023). Doximity reputation ranking is determined from the survey results of physician members of the American Society of Plastic Surgeons who have Doximity profiles. Physicians nominate up to 5 residency programs that they believe offer the best clinical training, and rankings are calculated based on the nominations. Doximity reputation rank is a well-known metric in the field of plastic surgery and has been used successfully in multiple recent publications relevant in the space.^7-9^ This ranking system has also been proven to affect how residency candidates view potential programs.^8,10^ Programs were excluded from data collection if they did not have at least 4 years of alumni data to analyze. The authors chose to use 4 years of data as a cutoff to maximize the number of programs included in the study, while providing sufficient data points for the analysis of each program.

### Data Analysis

Data were analyzed using SPSS statistical analysis software (IBM, Armonk, NY) and Microsoft excel (Redmond, WA). Descriptive statistics were used to analyze the trends in the dataset. Spearman's rank-order correlation analysis was used to assess the association between Doximity program rank and immediate postgraduation career paths. Spearman's correlation was also used to analyze relationships between secondary categorical variables. Fisher's exact test was used to determine significant associations between programs ranked in the top 25 and immediate postresidency career paths. Fisher's exact test was also used to determine significant associations between other secondary categorical variables (program size, program associated with a fellowship, program location, program size). The Fisher's exact test was the most appropriate statistical analysis for this dataset due to the smaller sample size and violation of the normal assumptions of the *χ*^2^ test (the expected frequency in each contingency was not sufficiently large). This method allowed appropriate evaluation of nonrandom associations between the outcomes of interest.

## RESULTS

A total of 88 programs were analyzed for this study. After review, 15 programs were excluded: 14 due to being relatively new programs with insufficient data for analysis and 1 for not having accessible data. A total of 1551 data points were collected from 73 programs between 2013 and 2022.

### Residency Programs Immediate Postgraduation Paths

Overall, PPCH was the most common immediate postgraduation path within all programs, with 37 programs having the majority of their graduates pursuing immediate PPCH positions between 2013 and 2022. University of California Irvine (UC Irvine), Emory University, Baylor College of Medicine, and University of Texas Southwestern Medical Center (UT Southwestern) had the highest number of graduates entering directly into PPCH. The University of Florida, UC Irvine, Emory University, and Loyola University had the highest percentage of their graduates entering PPCH based on total graduates between 2013 and 2022. Hand and microvascular fellowships were the next highest postgraduation pathway, with 11 programs having the highest percentage of graduates pursuing hand or microvascular specialties. The University of Michigan, Brigham and Women's Hospital/Harvard Medical School (Harvard), New York University (NYU), and University of San Francisco (UCSF) had the highest number of graduates entering microsurgery fellowship. Lehigh Valley Health Network, San Diego University, University of Michigan, and NYU had the highest percentage of their graduates entering microsurgery followship. For graduates entering hand fellowship, Harvard, University of Washington, Pittsburgh Medical Center, and Michigan State had the highest number of graduates entering, and Michigan State, Virginia Commonwealth University, and Wake Forest University had the highest percentage. For 6 programs, the highest percentage of graduates pursued aesthetic fellowship. University of Texas Southwestern, Wisconsin Medical College (Milwaukee), Duke University, and John Hopkins had the highest number of graduates entering aesthetic fellowships. Albany Medical College, Milwaukee, Zucker School of Medicine (Hofstra), and Dartmouth College had the highest percentage of graduates entering aesthetic fellowship. For graduates entering directly into academic practice, the University of Southern Florida (USF), Medstar Georgetown University, Harvard, Northwestern University, and University of Pennsylvania had the highest total number. Northwestern, USF, Medstar Georgetown, and Lahey Clinic had the highest percentage. Northwestern University is the only program that had the highest percentage of graduates pursuing immediate academic positions (Table 1). Graduates from Rutgers University, Stanford University, and Beth Israel Deaconess Medical Center pursued microvascular fellowship and hand fellowships at the highest rate equally. Graduates from University of Pennsylvania and Montefiore Medical Center entered microvascular fellowship and private/community-based practice at the highest rate equally. Graduates from Dartmouth College entered aesthetic fellowship and microvascular fellowship at the highest rate equally. Only 13 of the 73 included residency programs had graduates pursuing an immediate gender-affirming fellowship, with Mount Sinai having the highest percentage (13%). Eight programs had graduates who pursued a burn fellowship. Eight programs had graduates who entered military practice. Six programs had graduates who pursued a postgraduate career path specified as “other”.

**Table 1.** Top Immediate Postresidency Career Paths by Programs

Aesthetic fellowship	Burn fellowship	Craniofacial fellowship	Gender affirming	Hand fellowship	Microvascular fellowship	Academic practice	Private practice
Albany Medical College—53.8%^a^ (*T* = 13)	University of Southern California—7.9% (*T* = 38)	Chicago University—30% (*T* = 20)	Mount Sinai—13% (*T* = 23)	Michigan State University—58.8%^a^ (*T* = 17)	Lehigh Valley Health Network—71.4%^a^ (*T* = 7)	Lahey Clinic—26.7% (*T* = 15)	Florida University—77.8%^a^ (*T* = 9)
Wisconsin Medical College—42.9%^a^ (*T* = 21)	Virginia Commonwealth University—7.1% (T = 14)	UCLA School of Medicine—27.8%^a^ (T = 18)	Loma Linda University—6.7% (*T* = 15)	Yale Medical Center—45%^a^ (*T* = 20)	San Diego University—58.3%^a^ (*T* = 12)	Northwestern University—23.8%^a^ (*T* = 21)	Missouri University—75%^a^ (*T* = 16)
Dartmouth College—33.3%^a^ (*T* = 10)	University of North Carolina—7.1% (T = 14)	Zucker School of Medicine Hofstra/Northwell—25% (*T* = 12)	UCLA School of Medicine—5.6% (*T* = 18)	Virginia Commonwealth University—35.7%^a^ (*T* = 14)	Cooper University Hospital—57.1%^a^ (*T* = 7)	University of Southern Florida—23.3% (*T* = 30)	University of California Irvine—74.1%^a^ (*T* = 27)
Zucker School of Medicine Hofstra/Northwell—33.3%^a^ (*T* = 12)	Saint Louis University—4% (*T* = 25)	Yale Medical Center—25% (*T* = 20)	Brown University—5% (*T* = 20)	Washington University Saint Louis—34.8%^a^ (*T* = 23)	Michigan University—46.9%^a^ (*T* = 32)	Medstar Georgetown—21.4% (*T* = 28)	UT Houston—72.2%^a^ (*T* = 18)
Duke University—32.1%^a^ (*T* = 28)	Kansas University—4% (*T* = 25)	Penn State Medical College—22.2% (*T* = 18)	Indiana University—4.8% (*T* = 21)	Beth Israel Deaconess—33.3% (*T* = 9)	NYU—42.9%^a^ (*T* = 35)	Wright State University—20% (*T* = 10)	Loyola Medical Center—71.2% (*T* = 7)
Kentucky University—29.4% (*T* = 17)	Stanford University—3.7% (*T* = 27)	University of Massachusetts—21.1% (*T* = 19)	Montefiore Medical Center—4.5% (*T* = 22)	Penn State Medical College—33.3%^a^ (*T* = 18)	Chicago University—40%^a^ (*T* = 20)	Carilion Clinic Virginia Tech—20% *T* = 5)	Wright State—70%^a^ (*T* = 10)
Texas A&M College of Medicine—27.8% (*T* = 18)	University of San Francisco—3.6% (*T* = 28)	Minnesota University—20% (*T* = 10)	NYP Hospital (Columbia and Cornell)—4.5% (*T* = 22)	Stanford University—33.3% (*T* = 27)	University of San Francisco—39.3%^a^ (*T* = 34)	Dartmouth—20% (*T* = 10)	Emory University—68.8%^a^ (*T* = 32)
UT Southwestern—26.5% (*T* = 49)	Brigham and Women's Hospital (Harvard)—1.7% (*T* = 58)	Brown University—20% (*T* = 20)	Mayo Clinic Rochester—3.6% (*T* = 28)	University of Washington—32.4%^a^ (*T* = 34)	NYP Hospital (Columbia and Cornell)—36.4%^a^ (*T* = 22)	University of Pennsylvania—16.1% (*T* = 31)	University of South Carolina—66.7%^a^ (*T* = 12)
Cleveland Clinic Foundation—26.3% (*T* = 19)	n/a	Kansas University—20% (*T* = 25)	University of San Francisco—3.6% (*T* = 28)	University of Massachusetts 31.6%^a^ (*T* = 19)	Stanford University—33.3% (*T* = 27)	Baylor College of Medicine—15.6% (*T* = 32)	University of North Carolina—64.3%^a^ (*T* = 14)
John Hopkins University—24.2%^a^ (*T* = 33)	n/a	Carilion Clinic Virginia Tech—20% (*T* = 5)	University of Pennsylvania—3.2% (*T* = 31)	Virginia University—31.6%^a^ (*T* = 19)	Beth Israel Deaconess—33.3% (*T* = 9)	Colorado University—14.3% (*T* = 14)	Vanderbilt University—61.9%^a^ (*T* = 21)
Saint Louis University—24% (*T* = 25)	n/a	NYP Hospital (Columbia and Cornell)—18.2% (*T* = 22)	John Hopkins Medical Center—3% (*T* = 33)	Cleveland Clinic—31.6% (*T* = 19)	Dartmouth—33.3% (*T* = 10)	Vanderbilt University—14.3% (*T* = 21)	Oregon University—61.1%^a^ (*T* = 18)
Mayo Clinic Rochester—21.4%^a^ (*T* = 28)	n/a	Montefiore Medical Center—18.1% (*T* = 22)	University of Washington—2.9% (*T* = 34)	Brown University—30%^a^ (*T* = 20)	Washington University Saint Louis—30.4% (*T* = 23)	Indiana University—14.3% (*T* = 21)	Loma Linda—60%^a^ (*T* = 15)
University of Massachusetts—21.1% (*T* = 19)	n/a	University of San Francisco—17.9% (*T* = 28)	Pittsburgh Medical Center—2.1% (*T* = 47)	Southern Illinois University—29.4% (*T* = 17)	Utah University—28.6%^a^ (*T* = 21)	Emory University—12.5% (T = 32)	Colorado University—57.1%^a^ (*T* = 14)
Ohio State—20.8% (*T* = 24)	n/a	Mount Sinai—17.4% (*T* = 23)	n/a	Lehigh Valley Health—28.6% (*T* = 7)	Montefiore Medical Center—27.3% (*T* = 22)	Saint Louis University—12% (T = 25)	Mississippi University—55.6%^a^ (*T* = 18)
LSU—18.8% (*T* = 16)	n/a	Rutgers University—15.8% (*T* = 19)	n/a	Texas Medical Branch—26.5% (*T* = 34)	Texas Medical Branch—26.5% (*T* = 34)	Mayo Clinic Rochester—10.7% (*T* = 28)	Baylor Medical Center—50%^a^ (*T* = 32)

*T*, total number of graduates from 2013 to 2022 by which percentages are based. ^a^The most common postresidency career path of the program.

### Trends in Immediate Postgraduation Paths Over Time

**Figure 1.**
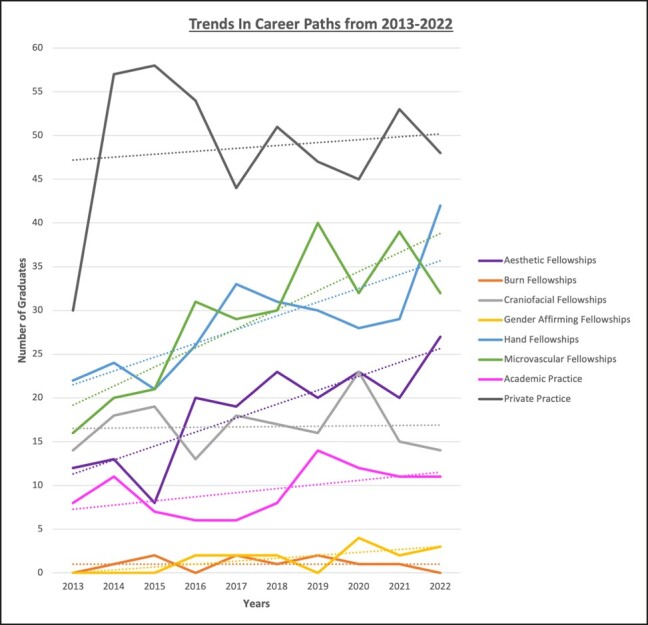
A line graph demonstrating postresidency graduation paths from 2013 to 2022. The dotted line of the same color represents the trend line over time. This is representative of the 1551 graduates included in this study.

**Figure 2.**
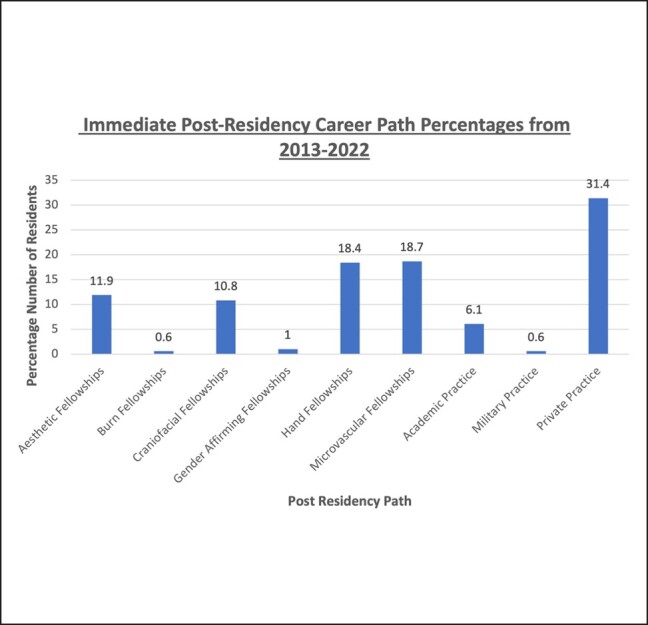
A bar graph with the percentage of each postresidency career path from 2013 to 2022. This is representative of the 1551 graduates included in the study.

Overall, there has been an increasing trend in plastic surgery residents pursuing fellowships, particularly in hand, microvascular, and aesthetic surgery. Between 2013 and 2022, microsurgery and aesthetic fellowships have seen the strongest growth, with slopes of 2.181 and 1.594, respectively. Residents entering directly into gender-affirming fellowships, PPCH, and academic practice have seen a moderate increase over time slopes of 0.333, 0.333, and 0.473, respectively. Residents pursuing craniofacial fellowships have remained stable over the past 9 years represented by a slope of 0.042. Between 2013 and 2015 of the 390 graduates, PPCH accounted for 40.1% of postresidency career paths compared with a combined 43% between aesthetic, hand, microvascular, and gender-affirming fellowships. Between 2020 and 2022 of the 517 graduates, PPCH accounted for 28.3% of postresidency career paths, compared with a combined 55% between aesthetic, hand, microvascular, and gender-affirming fellowships (see the full trend graph in Figure 1). Among the 1551 graduates from 2013 to 2022, the mean number pursuing PPCH, microsurgery fellowship, hand fellowship, aesthetic fellowship, and academic practice was 33.6, 18.6, 17.6, 12.2, and 5.7, respectively (Figure 2).

### Secondary Variable Analysis

Based on the Doximity reputation ranking system, lower-ranked programs were significantly correlated to a higher percentage of graduates entering PPCH (*P* = .005), with a Spearman's rho (*ρ*) correlation of 0.327, representing a moderately weak association. A moderately strong association was found between program rank and associated fellowship programs (*ρ* = 0.545, *P* < .001). Programs in the top 25 Doximity reputation rank had a significant association with craniofacial fellowships (*P* = .05), microvascular fellowship (*P* = .021), and immediate academic appointments (*P* = .011). Programs that had associated independent plastic surgery residents were significantly associated with immediate academic appointments (*P* = .019). Programs in the Northeast were significantly associated with residents pursuing gender-affirming fellowship (*P* = .047).

## DISCUSSION

The field of plastic surgery continues to see tremendous growth and expansion. The complexity of procedures is evolving and requires more training in specific areas. Plastic surgery residents have reported that procedure complexity and unfamiliarity with procedures are the main limiting factors of feeling confident to perform procedures autonomously.^4^ This may be one element contributing to the increasing trends of residents pursuing postgraduate fellowships. There is also previous evidence demonstrating a lack of training in certain specific areas among residency trainees, including aesthetic surgery, craniofacial surgery, and gender-affirming care.^4,11-14^ There are many factors that could play a role in plastic surgery residency graduates feeling unprepared to practice fully autonomously in all areas of the field. These factors include resident hour restrictions, stricter hospital regulations (eg, required attending presence in the operating room), financial limitations (eg, regulations on reimbursement), patients’ concerns with resident participation in care, and increased focus on patient safety.^4^ Recent evidence discovered that up to 66% of plastic surgery attendings felt there had been a decrease in resident autonomy compared with residents 10 years ago.^4^ This fits well with the overall trend of increasing pursuit of fellowships over the last 10 years found in the results of this paper.

Aesthetic surgery is an area that residents have consistently reported feeling unprepared to practice autonomously.^4,11,12^ Aesthetic surgery has seen a rise in demand over the past few years. According to the Aesthetic Society, in 2021, surgical procedures increased 54% compared with 2020.^15^ This makes specializing in aesthetic surgery desirable for graduates, as more demand means more chances to build volume, leading to a successful practice. Residencies have been adapting to help better residents feel more competent to meet the demands of the aesthetic patient. An important change to furthering this goal was increasing the required number of aesthetic cases needed for graduation.^12^ Recently, residents have reported feeling more confident in their ability to perform aesthetic procedures, but still report the need for overall improvement in aesthetic training.^4,11^ Newer and more niche procedures are gaining popularity among patients.^15^ Procedures such as gluteal augmentation, hair transplant, and endoscopic techniques may be difficult to incorporate into the resident curriculum and may require further fellowship training to gain competency.^11,15^ This correlates with information obtained in our data analysis, with aesthetic fellowships showing the second highest positive trend in the past 10 years (slope = 1.594).

Craniofacial surgery is another area of plastic surgery residency where graduates feel unprepared to perform at a high level.^4,13^ This is likely because these low-prevalence congenital procedures are frequently referred to high-volume centers, resulting in residents having less exposure to these procedures and to different facets of longitudinal care. This correlates with evidence found in this paper, with craniofacial fellowships remaining relatively constant over the last 10 years. Interestingly, there was a significant association with residents pursuing craniofacial fellowships and programs ranked in the top 25 by Doximity reputation.

Gender-affirming care is another area that is growing in plastic surgery. Approximately 1 million Americans identify themselves as transgender, between 20% and 40% elect to undergo gender-affirming surgery, and that number is expected to increase dramatically.^14^ Plastic surgeons are considered the most appropriate surgical specialty to refer these patients to, demonstrating the importance of training in this area.^14^ While this is a relatively new trend, research in this paper did show a positive trend in plastic surgery graduates pursuing gender-affirming fellowships. Interestingly, most surveyed plastic surgery residency directors felt that residents were well trained in gender-affirming care; however, when assessing different expectations of gender-affirming surgery, residency directors report high resident exposure to top/chest surgery (∼91%) and low exposure to bottom/genital surgery (∼28%).^14^ Facial surgery is also an essential component of gender-affirming care, which presents a challenge to residents, as this is a commonly reported area of difficulty.^4-6,9,11,12,14^ Indicating if residents want to be able to offer all aspects of gender-affirming surgery, fellowship training may be beneficial. Of note, Mount Sinai was the only residency program that had multiple graduates pursuing gender-affirming fellowship, which could make it attractive to students interested in transgender care. Notably, Mount Sinai offers a gender-affirming fellowship, which may play a role in the increased number of residency graduates pursuing further gender-affirming training. The authors did not investigate at what specific programs residents completed their fellowships. An interesting question would be if these residents were more likely to complete fellowship at their home residency program vs going on to complete fellowship at a different institution. The geographical location of the residency program was also a factor for gender-affirming fellowships. Programs in the Northeast were significantly associated with residents pursuing gender-affirming fellowships (*P* = .0047). Outside of the Northeast, the only other states that had programs with residents pursuing gender-affirming fellowships were California, Minnesota, Washington, and Indiana. Medical students with strong ambition to pursue transgender care may look to attend programs in certain geographical areas to increase exposure and improve the likelihood of successfully matching into gender-affirming fellowship if desired.

The pursuit of immediate academic appointment saw a moderate increase (slope = 0.473) over the last 10 years; however, those interested in pursuing academic medicine may be choosing to pursue a postgraduate fellowship first. A recent study revealed that approximately 70% of academic plastic surgeons completed fellowship training.^16^ In that study, a within-group analysis showed that assistant professors, associate professors, and residency directors were the most likely positions to have completed a fellowship.^16^ The majority of full professors had completed fellowships (63%), but they were significantly less likely to have completed their fellowships compared with assistant and associate professors.^16^ This correlates with the data in our study. Assistant and associate professors are typically newer faculty compared with full-time professors and therefore more likely to have pursued fellowship training over the last 10 years.

A significant correlation in our study was between lower-ranked programs and graduates entering PPCH (*P* = .005, *ρ* = 0.327). This may be related to several factors. Of the criteria reported in the literature that make candidates more likely to be selected for postgraduate fellowship, 2 of the factors are letters of recommendation from those in the specialty and research productivity.^17-20^ Research productivity is among the criteria used for ranking residency training programs, which could explain why lower-ranked programs were more likely to enter PPCH rather than fellowship. Another significant correlation in our study was for higher-ranked residency programs to have associated fellowship programs (*P* < .001, *ρ* = 0.545). Graduates of programs with associated fellowships have more opportunities to interact with surgeons in those subspecialties, thereby increasing the chances of obtaining strong letters of recommendation in those fields. Another factor possibly associated with academic appointment is the prestige of the program that one graduates from. Our study revealed a significant association between programs ranked in the top 25 and immediate academic appointment (*P* = .011). This suggests that residents with an interest in academic medicine may have an increased likelihood of securing academic positions directly after graduation from programs historically perceived as being more prestigious without having to complete a fellowship.

An alternative perspective on why residents graduating from higher-ranked programs are less likely to immediately enter PPCH could be that those from programs without associated fellowships feel more confident in their readiness to enter directly in practice. Surveys of surgical residents have revealed possible negative impacts of having fellows in the same department.^21,22^ The most common reported complaints are that residents experience decreased operative volumes, and that learning from fellows during an operation is not as beneficial as working with attending faculty independently.^21,22^ Since fellowship programs are more likely to be connected to higher-ranked residency programs, this may explain the stronger association observed between comparatively lower-ranked programs and immediate entry into PPCH. The positive association between the top 25 residency programs and residents pursuing craniofacial fellowship may also be explained by this. With residents already reporting decreased confidence with craniofacial surgical management, losing operative experience to craniofacial fellows may amplify that reduced confidence.^4,13^

Microvascular surgery is arguably the most rapidly developing field in plastic surgery in large part due to advancements in magnification instruments, surgical devices, perforator vessel dissection techniques, and vessel imaging.^23^ There is a broad range of applications for microvascular surgery, including breast reconstruction, extremity salvage, gender-affirmation surgery, and lymphatic surgery.^23^ While there are requirements for microsurgery experience in residency, fellowships offer heavy volume of microvascular cases, increased exposure to complex cases, improved surgical autonomy, and ability to subspecialize further within the microvascular field.^23^ This may explain why our data revealed that over the last 10 years, graduates pursuing microvascular fellowships had the highest increase compared with any other specialty and were the second leading immediate postgraduate pathway after PPCH. Our study also demonstrated that programs ranked in the top 25 were significantly associated with residents pursuing microvascular fellowship (*P* = .021). Accordingly, prospective plastic surgery applicants with the strong desire to pursue microvascular surgery may look to enroll in these top 25 programs.

### Limitations

There are several limitations in our study that should be acknowledged. The data relied on open-source institutional listings available online. Most residency programs had an alumni section that listed graduates and their immediate postgraduation path. For programs that did not list this, Doximity was the primary source of information in determining the postgraduation pathway for alumni. To maximize accuracy, information from programs without an alumni page was cross-checked with either the program-specific plastic surgery Instagram page, personal website biographies, or LinkedIn. Data were collected from the years 2013 to 2022. Not all programs listed alumni data for that entire period, and Doximity was used to fill the gaps. There are limitations to Doximity because it requires that a person has a Doximity account to obtain their alumni information; thus, there were gaps in some of the programs' data. This limitation was minimized by reporting both the percentage and the total number of postresidency career paths by year. Another limitation may be the use of Doximity reputation rank. Doximity reputation rank is based on the subjective opinions of certified members of the American Board of Plastic Surgery. Because it is not based on objective measures, there may be bias within the rankings. This is an inherent problem with ranking systems. There are a limited number of systems that rank plastic surgery residency programs strictly by objective measures. A previous study by Boyd et al created a ranking system of programs based on the academic achievement of faculty members.^24^ Doximity offers an alternative ranking system that lists programs in order of research output. We recognize that the metrics used in all 3 of these ranking systems do not objectively reflect the residency training experience. Interestingly, however, despite the differences in the data used for these ranking systems, there is an overlap of programs across these lists, and programs ranked within the top 25 largely remain consistent across different ranking systems. A side-by-side comparison of these ranks is given in Table 2. One more important limitation is that no independent-only plastic surgery programs were included in this study; however, there were plastic surgery programs that transitioned from independent to integrated during the data collection period. To minimize this limitation, data were collected from both independent and integrated graduates and were analyzed together. Lastly, for statistical analysis, a multivariate linear regression may have been employed to determine significant relationships between the secondary variables and the immediate postresidency career paths. This analysis would control for confounding variables. However, because of the data's small sample size and the violation of normality in the dependent variables, the multivariate linear regression model would not be valid; therefore, the Fisher's exact test was used to assess significant associations between variables.

**Table 2.** Comparison of Different Top 25 Plastic Surgery Residency Program Lists

Rank order	Doximity research output (2023)	Doximity reputation rank (2023)	Faculty academic achievement (2021)
1	Harvard^a^	University of Washington^a^	Harvard^a^
2	University of Michigan^a^	University of Pittsburgh^a^	Stanford University^a^
3	John Hopkins University^a^	University of Michigan^a^	University of Michigan^a^
4	Duke University^a^	NYU^a^	UCLA^a^
5	University of Pittsburgh^a^	UT Southwestern^a^	University of Pennsylvania^a^
6	NYU^a^	John Hopkins University^a^	Washington University in St Louis^b^
7	Stanford University^a^	University of Pennsylvania^a^	NYU^a^
8	University of Wisconsin^b^	Harvard^a^	John Hopkins University^a^
9	Northwestern University^a^	Stanford University^a^	University of Pittsburgh^a^
10	University of Washington^a^	University of Wisconsin^b^	Duke University^a^
11	Vanderbilt University	University of Southern California^b^	Ohio State University^b^
12	University of Pennsylvania^a^	Mayo Clinic Rochester	University of Cincinnati
13	UCLA^a^	UCLA^a^	UC San Francisco^b^
14	Beth Israel Deaconess^b^	Emory University^b^	Emory University^b^
15	University of Chicago^a^	Baylor College^a^	Cleveland Clinic
16	UT Southwestern^a^	Northwestern University^a^	University of Southern California^b^
17	UC San Francisco^b^	Washington University in St Louis^b^	Northwestern University^a^
18	University of Virginia	Duke University^a^	Yale^a^
19	Yale^a^	Wisconsin Medical Center	Baylor College^a^
20	Brown University	Georgetown Medstar	University of Chicago^a^
21	UC Davis	University of Chicago^a^	UT Southwestern^a^
22	Baylor College^a^	Yale^a^	University of Washington^a^
23	New York Presbyterian	Texas A&M^b^	University of Florida
24	UC Irvine	San Diego University	Texas A&M^b^
25	Ohio State University^b^	Beth Israel Deaconess^b^	University of Kentucky

^a^Programs in all 3 lists.

^b^Programs in 2 lists.

## CONCLUSIONS

Our data suggest that immediate postgraduate career paths are becoming more diverse among residency programs. Most postresidency paths are divided between PPCH, microvascular fellowship, and hand fellowship. The pursuit of microvascular, aesthetic, and hand fellowships demonstrates the largest increase for plastic surgery graduates, while craniofacial fellowships have remained relatively consistent. Selecting gender-affirming fellowships demonstrated a positive trend, but overall, the total number of graduates entering this pathway is low. As the need for transgender care continues to rise, this upward trend may continue. Burn fellowships showed the lowest overall totals and did not have any increased trends over the last 10 years. Interestingly, previous research shows that most residents feel comfortable treating patients with burn injuries, which could play a role in the small number of residents pursuing burn fellowships.^4,25^

The evidence presented here can be helpful for prospective plastic surgery candidates and residency programs with the changing landscape and the evolving needs of the field. As medical students become exposed to plastic surgery, their evolving interests and aspirations may guide their choices when ranking their residency programs. Understanding the career paths and specializations pursued by graduates can provide valuable insights for both prospective residents and residency programs. This information can assist students in identifying the programs that align best with their individual career goals and interests, enabling them to make informed decisions about their future in plastic surgery. Equally, residency programs can use this information to attract and recruit students who are likely to thrive in specific areas of the field. Future research should aim to investigate whether applicants consider the postresidency career paths of program residents as a significant factor in their program ranking decisions and to what extent this consideration influences their final ranking selections.
